# Epigenetic Induction of Definitive and Pancreatic Endoderm Cell Fate in Human Fibroblasts

**DOI:** 10.1155/2016/7654321

**Published:** 2016-06-15

**Authors:** Rangarajan Sambathkumar, Eric Kalo, Rob Van Rossom, Marijke M. Faas, Paul de Vos, Catherine M. Verfaillie

**Affiliations:** ^1^Interdepartmental Stem Cell Institute, Department of Development and Regeneration, Stem Cell Biology and Embryology, KU Leuven, 3000 Leuven, Belgium; ^2^University Medical Center Groningen (UMCG), Pathology and Medical Biology, Section of Immunoendocrinology, University of Groningen, Hanzeplein 1, EA 11, 9713 GZ Groningen, Netherlands

## Abstract

Reprogramming can occur by the introduction of key transcription factors (TFs) as well as by epigenetic changes. We demonstrated that histone deacetylase inhibitor (HDACi) Trichostatin A (TSA) combined with a chromatin remodeling medium (CRM) induced expression of a number of definitive endoderm and early and late pancreatic marker genes. When CRM was omitted, endoderm/pancreatic marker genes were not induced. Furthermore, treatment with DNA methyltransferase inhibitor (DNMTi) 5-azacytidine (5AZA) CRM did not affect gene expression changes, and when 5AZA was combined with TSA, no further increase in gene expression of endoderm, pancreatic endoderm, and endocrine markers was seen over levels induced with TSA alone. Interestingly, TSA-CRM did not affect expression of pluripotency and hepatocyte genes but induced some mesoderm transcripts. Upon removal of TSA-CRM, the endoderm/pancreatic gene expression profile returned to baseline. Our findings underscore the role epigenetic modification in transdifferentiation of one somatic cell into another. However, full reprogramming of fibroblasts to *β*-cells will require combination of this approach with TF overexpression and/or culture of the partially reprogrammed cells under *β*-cell specific conditions.

## 1. Introduction

Type-1 diabetes mellitus (DM-I) is a severe metabolic disease that affects millions of people worldwide. It involves complete loss of functional insulin secreting *β*-cells in the pancreas due to autoimmune destruction. DM-1 leads to hyperglycemia, which can be treated with insulin injections. However, in the long term, DM-1 leads to micro- and macrovascular, cardiovascular, neuronal, renal, and ocular complications due to intermittent hyperglycemia. Replacement of the destroyed *β*-cells is the only curative treatment. However, limited numbers of available donor organs and immunological issues restrict whole pancreas or islet transplantation [1, 2]. An alternative source for human cadaveric islets is generating insulin-producing *β*-cells from stem cells and/or somatic cells. However, the challenge in this area is to identify an adequate stem or progenitor cells and mechanisms to create a safe source of mature insulin-producing *β*-cells from such cells.

Over the last decade, several studies have demonstrated that it is possible to generate functional *β*-cells from human embryonic stem cells (hESCs) by culturing the cells in conditions that mimic* in vivo* pancreatic development [3–6]. With the advent of induced pluripotent stem cells (iPSCs) technology, developed by the Yamanaka team [7, 8], it has now also become possible to create Human Leukocyte Antigen- (HLA-) identical *β*-cells to treat DM-I. However, the pluripotent nature of ESCs and iPSCs leaves the possibility for teratoma formation [6, 9], if full differentiation towards *β*-cells is not achieved. An alternative approach is to transdifferentiate somatic cells into insulin-producing *β*-cells without passing through a pluripotent state, via ectopic expression of defined TFs and culture in supportive medium. A number of studies demonstrated in rodents that introduction of a single (*PDX1*) or group of TFs (*PDX1, NGN3, and MAFA*) can transdifferentiate hepatocytes, intestinal cells, exocrine acinar or ductal pancreatic cells, or endocrine *α*-cells, thereby reprogramming these non-*β*-cell endodermal cells into *β*-cells [10–17]. In addition, Li et al. created endodermal progenitor cells by transiently overexpressing* OCT4*,* SOX2, KLF4*, and* CMYC* (OKSM) in mouse embryonic fibroblasts (MEFs) combined with small molecule epigenetic modifiers, which could subsequently be converted to *β*-cells [18]. Several recent studies have treated human or swine fibroblasts [19–21], mesenchymal stem cells [22], or rat liver stem cells [23], with epigenetic modifying molecules (DNA methyltransferase inhibitor and/or histone deacetylase inhibitor) in CRM followed by culture under *β*-cell specifying conditions. This combined treatment resulted in the generation of endocrine pancreatic *β*-cells that reversed hyperglycemia in immunodeficient mice. Moreover, epigenetic modification also induced NGN3 expression and endocrine differentiation of the PNAC-1 human ductal cell line [24]. However, many studies reported that TSA itself could induce chromatin changes without the presence of chromatin remodeling medium (CRM). For instance, using fluorescence anisotropy imaging and fluorescence recovery after photobleaching (FRAP) and fluorescence correlation spectroscopy (FCS), it was demonstrated that TSA induced histone protein dynamics and expression in HeLa cells by increasing the euchromatin fraction and increasing core acetylation patterns, phosphorylation patterns, and nuclear volume [25]. Similarly, TSA induces histone acetylation and reversible decondensation of interphase chromatin structure in HeLa cells, as demonstrated by image correlation spectroscopy (ICS) and spatially resolved scaling analysis (SRSA) methods [26]. Furthermore, treatment with TSA and VPA (valproic acid) increased the active chromatin marks, such as H3K9ac and H3K4me2 abundance, which might lead to chromatin decondensation in human hepatocellular carcinoma (HepG2) and NIH 3T3 cells [27, 28].

We here optimized the exposure of human fibroblasts to epigenetic modifiers to convert them to endoderm and pancreatic endocrine progenitors. We demonstrated that culture of adult human and foreskin fibroblasts with TSA combined with a chromatin remodeling medium (CRM) induces expression of endoderm and pancreatic endoderm genes but that this is a transient phenomenon. Hence, further maintenance in *β*-cell-specifying conditions with or without forced expression of exogenous TFs will be needed to permanently convert fibroblasts to *β*-cells.

## 2. Materials and Methods

### 2.1. Culture of BJ1 Human Fibroblasts

Human BJ1 adult fibroblasts were cultured in 90% DMEM-F12 + HEPES (Life Technologies, NY, USA), 10% Fetal Bovine Serum (FBS, HyClone, USA), and 1% Penicillin/Streptomycin (Life Technologies). Cells were maintained under normoxic conditions, 37°C temperature, and 5% CO_2_.

### 2.2. Isolation and Culture of Primary Human Fibroblasts

A skin biopsy from healthy volunteer/nondiabetic donor was obtained following informed consent and with approval from the ethical committee of University of KU Leuven. Fibroblasts were isolated in minimum DMEM supplemented with 20% FBS. After four passages, fibroblasts were frozen in liquid nitrogen in several aliquots. After thawing cells were grown in 90% DMDM HG + Glutamax (Life Technologies), supplemented with 10% FBS and 1% Penicillin/Streptomycin (Life Technologies). Cells were maintained under normoxic conditions, 37°C temperature, and 5% CO_2_.

### 2.3. Reprogramming Conditions

Chromatin remodeling medium (CRM) consisted of 10% knockout (KO) serum, KO-DMEM, 50 mM *β*-mercaptoethanol, 1% nonessential amino acids, 1% B27 supplement, 2 mM L-glutamine, and 2% N2 supplement, all from Life Technologies, and 20 ng/mL fibroblast growth factor (Peprotech, USA), 20 ng/mL epidermal growth factor (R&D Systems, MN, USA), 1000 U/mL Penicillin/Streptomycin (Life Technologies), and 100 nM L-Ascorbic Acid (Sigma Aldrich). Cells were plated in 6-well plate (VWR, MA, USA) and exposed to 3 *μ*M or 5 *μ*M 5AZA (Sigma Aldrich, dissolved in DMSO) in CRM medium for 48 hours, with medium change at 24 h. For treatment with Trichostatin A (TSA), ready-made solution 5 mM (Sigma Aldrich), cells were exposed to various concentrations between 100 nM and 100 *μ*M for 24 hours. Untreated fibroblasts and DMSO treated fibroblasts cultured in CRM were used as controls.

### 2.4. RNA Isolation, cDNA Synthesis, and Quantitative Real-Time Polymerase Chain Reaction (qRT-PCR)

Total RNA was extracted using GenElute*™* Mammalian Total RNA Miniprep Kit (Sigma Aldrich); for sample size < 10^5^ cells, ZR RNA Microprep*™* CA, USA, was used and cDNA was synthesized from 1 *μ*g total RNA using superscript III reverse transcriptase (Life Technologies) both according to manufacturer's protocol. For qPCR, the cDNA underwent 40 rounds of amplification cycles on a ViiA*™* 7 Real-Time PCR System (Applied Biosystems) as follows: 40 cycles of a 2-step PCR (95°C for 15 sec, 60°C for 45 sec) after initial denaturation (50°C for 2 min, 95°C for 2 min) using specific primers, Platinum® SyBR® Green qPCR SuperMix-UDG (Life Technologies), and 2 *μ*L cDNA. For normalization purposes,* GAPDH* (glyceraldehyde 3-phosphate dehydrogenase) was used as a housekeeping control and results are shown in relative expression to* GAPDH*. All primers were synthesized at IDT Technologies, Belgium. The cycle threshold value > 40 is considered undetectable and calculated as Ct of 40. A list of the primers can be found in Table 1.

### 2.5. Statistical Analysis

Parametric distribution of data points was confirmed using the Kolmogorov-Smirnov test. Comparisons between two groups were analyzed using an unpaired 2-tailed Student's *t*-test. ^*∗*^ *p* < 0.05, ^*∗∗*^ *p* < 0.01, and ^*∗∗∗*^ *p* < 0.001 were considered significant. Data are shown as mean and error bars represent standard error of mean (SEM) of minimum three independent experiments. All results were analyzed using Graph Pad prism 6 software.

## 3. Results

### 3.1. Treatment of Human Primary Adult and Foreskin BJ1 Fibroblasts with TSA Induced Definitive Endoderm and Pancreatic Endocrine Genes but Only Transiently

**Figure 1:**
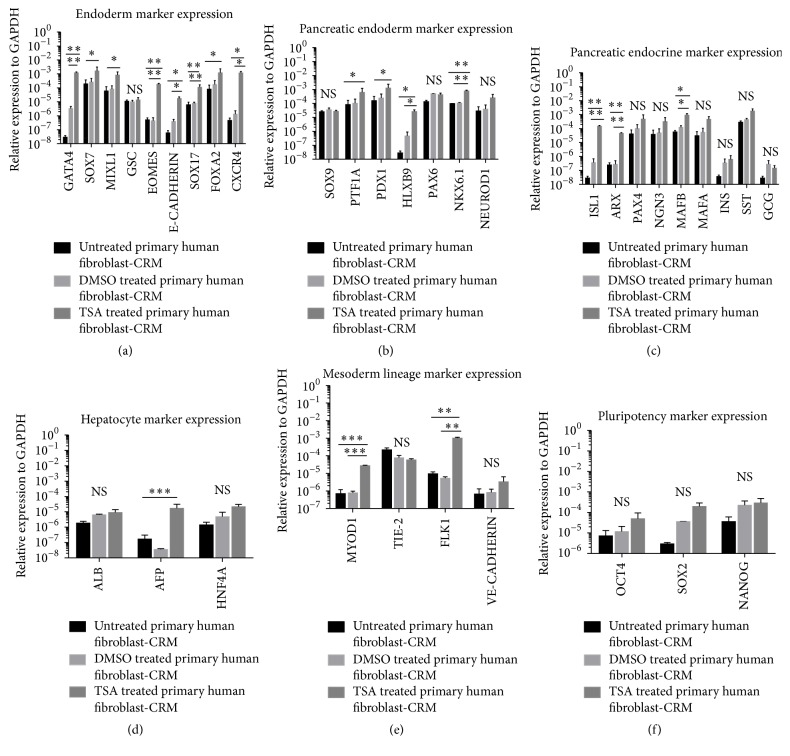
Trichostatin A (TSA) treatment of primary adult fibroblasts induces transient definitive endoderm and pancreatic endoderm markers. (a) qRT-PCR analysis demonstrated induction of endodermal genes (*GATA4, SOX7, MIXL1, EOMES, E-CADHERIN, SOX17, FOXA2*, and* CXCR4* but not* GSC*) in TSA-CRM treated primary human fibroblast. (b) qRT-PCR analysis demonstrated induction of pancreatic endoderm genes (*PTF1A, PDX1, HLXB9*, and* NKX6.1* but not* SOX9, PAX6*, and* NEUROD1*) in TSA-CRM treated primary human fibroblast. (c) qRT-PCR analysis demonstrated induction of pancreatic endocrine genes (*ISL1, ARX*, and* MAFB* but not* PAX4, NGN3, MAFA, INS, SST*, and* GCG*) in TSA-CRM treated primary human fibroblast. (d) qRT-PCR analysis demonstrated that hepatocyte genes (*ALB* and* HNF4A*) were not induced except for* AFP*. (e) qRT-PCR analysis demonstrated induction of mesoderm lineage genes (*MYOD1* and* FLK1* but not* TIE-2* and* VE-CADHERIN*) in TSA-CRM treated primary human fibroblast. (f) qRT-PCR analysis demonstrated no induction of pluripotency genes (*OCT4, SOX2*, and* NANOG*) expression in TSA-CRM treated primary human fibroblast. Black bar, CRM treated primary human fibroblast cells; light grey bar, DMSO-CRM primary human fibroblast treated cells; dark grey bar, 100 *μ*M TSA-CRM treated primary human fibroblast cells. Gene expression is shown relative to the housekeeping gene* GAPDH*. Data represent the mean ± SEM (standard error of mean) of three independent experiments. Statistical significance tests were performed between TSA treated versus untreated fibroblast and TSA treated versus DMSO treated fibroblast. ^*∗*^ *p* < 0.05, ^*∗∗*^ *p* < 0.01, and ^*∗∗∗*^ *p* < 0.001 by unpaired 2-tailed Student's *t*-test. NS, not significant.

We initially tested the effect of addition of the HDAC inhibitor, TSA for 24 h, on human primary adult and BJ1 foreskin fibroblasts, cultured in chromatin remodeling medium (CRM). We then assessed levels of transcripts of definitive endoderm and early and late pancreatic endocrine markers, as well as hepatocyte, skeletal muscle, endothelium, and pluripotency marker genes. TSA/CRM significantly induced the expression of the early endoderm marker genes,* GATA4, EOMES, E-CADHERIN, SOX17, FOXA2*, and* CXCR4*, in both cell populations, while induction of* SOX7* and* MIXL1* was only seen in primary human fibroblasts (Figure 1(a) and Figure S1A in Supplementary Material available online at http://dx.doi.org/10.1155/2016/7654321). In addition, expression of the early and late pancreatic progenitor and endocrine marker genes,* PTF1A, HLXB9, NKX6.1, ISL1, ARX*, and* MAFB*, was observed in both cell populations, while* PDX1* expression was only detected in primary human fibroblasts treated cells (Figures 1(b)-1(c) and Figure S1B-C). Transcripts for mature endocrine pancreatic cells including* PAX4, NGN3, INS, GCG*, and* SST* were however not increased following TSA/CRM culture (Figure 1(c) and Figure S1C). Transcripts for the hepatocyte marker genes,* ALB, AFP*, and* HNF4A* (Figure 1(d) and Figure S1D), were not expressed. We also assessed the effect of TSA/CRM on expression of mesodermal lineage transcripts and found an increase in* MYOD1* and* FLK1* expression but not the endothelium marker genes* TIE2* and* VE-CADHERIN* (Figure 1(e) and Figure S1E). Pluripotency marker genes* OCT4, SOX2*, and* NANOG* (Figure 1(f) and Figure S1F) were not induced. When the remodeling medium was removed, expression of the pancreatic endodermal genes was not maintained.

### 3.2. Combined Treatment of Fibroblasts with 5AZA and TSA Did Not Result in Further Increased Expression of Definitive Endoderm and Pancreatic Endocrine Genes

To test if combination of DNA methylation modification and inhibition of HDAC would induce a significant transdifferentiation of fibroblasts to endocrine pancreas, we next cultured primary adult fibroblasts and BJ1 fibroblast cells in CRM supplemented for 2 days with 3 or 5 *μ*M 5AZA for 48 hours, followed by TSA for 24 hours. However, as significant cell death was seen with 5AZA at 5 *μ*M or more, studies were done using 3 *μ*M 5AZA. Treatment for 2 days with 5AZA induced subtle morphological changes. In comparison with the typical elongated morphology of untreated fibroblasts, 5AZA treated cells had a more rounded shape without long processes. 5AZA also decreased cell proliferation, which was analyzed by cell counting (data not shown). Following treatment with TSA on day 3, the cells appeared more flattened with granular cytoplasm and larger nuclei possibly due to more relaxed chromatin structure. Cell proliferation remained stable upon exposure to TSA.

**Figure 2:**
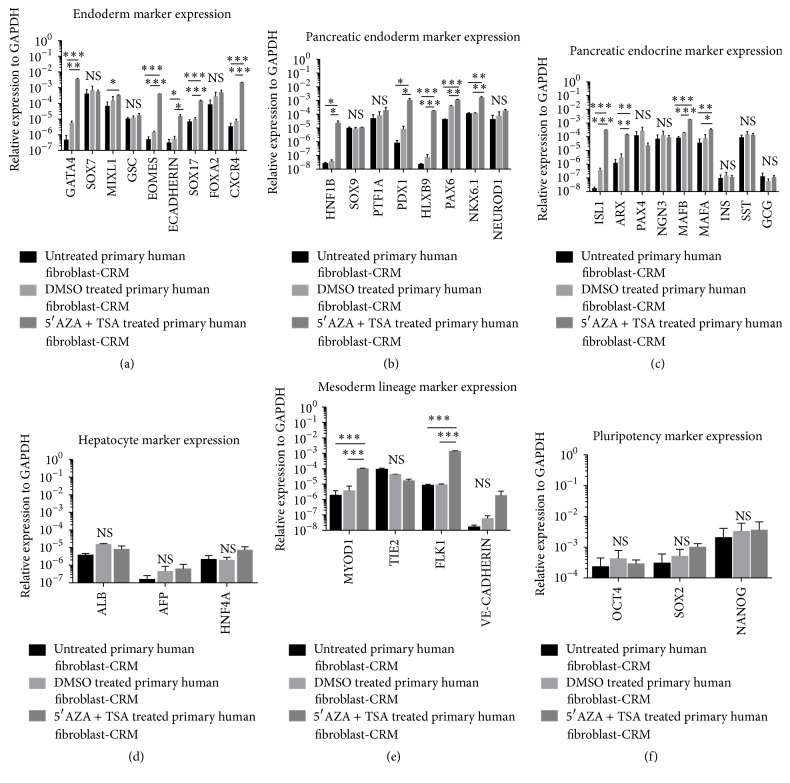
5-Azacytidine (5AZA) and Trichostatin A (TSA) treatment of primary adult fibroblast induces transient definitive endoderm and pancreatic endoderm markers. (a) qRT-PCR analysis demonstrated induction of endodermal genes (*GATA4, MIXL1, EOMES, E-CADHERIN, SOX17*, and* CXCR4* but not* SOX7, GSC*, and* FOXA2*) in 5AZA + TSA-CRM treated primary human fibroblast cells. (b) qRT-PCR analysis demonstrated induction of pancreatic endoderm genes (*HNF1B, PDX1, HLXB9, PAX6*, and* NKX6.1* but not* SOX9, PTF1A*, and* NEUROD1*) in 5AZA + TSA-CRM treated primary human fibroblast cells. (c) qRT-PCR analysis demonstrated induction of the pancreatic endocrine genes (*ISL1, ARX, MAFB*, and* MAFA* but not* PAX4, NGN3, INS, SST*, and* GCG*) in 5AZA + TSA-CRM treated primary human fibroblast cells. (d) qRT-PCR analysis demonstrated no induction of hepatocyte genes (*ALB, AFP*, and* HNF4A*) in 5AZA + TSA-CRM treated primary human fibroblast cells. (e) qRT-PCR analysis demonstrated induction of mesoderm lineage genes (*MYOD1* and* FLK1* but not* TIE-2* and* VE-CADHERIN*) in 5AZA + TSA-CRM treated primary human fibroblast cells. (f) qRT-PCR analysis demonstrated no induction of pluripotency genes (*OCT4, SOX2*, and* NANOG*) in 5AZA + TSA-CRM treated primary human fibroblast cells. Black bar, CRM treated primary human fibroblast cells; light grey bar, DMSO-CRM treated primary human fibroblast cells; dark grey bar, 3 *μ*M 5AZA-CRM followed by 100 *μ*M TSA-CRM treated primary human fibroblast cells. Gene expression is shown relative to the housekeeping gene* GAPDH*. Data represent the mean ± SEM (standard error of mean) of three independent experiments. Statistical significance tests were performed between 5AZA + TSA treated versus untreated fibroblast and 5AZA + TSA treated versus DMSO treated fibroblast. ^*∗*^ *p* < 0.05, ^*∗∗*^ *p* < 0.01, and ^*∗∗∗*^ *p* < 0.001 by unpaired 2-tailed Student's *t*-test. NS, not significant.

We next assessed the effect of 5AZA combined with TSA on transcript expression as described for TSA only studies above. By contrast, following treatment with 5AZA followed by TSA, endodermal early and late pancreatic endocrine progenitor marker genes were induced in both primary and BJ1 foreskin fibroblasts (Figures 2(a)–2(c) and Figures  S2A–C). However, as with TSA only in this case also, mature pancreatic endocrine marker genes were not induced (Figure 2(c) and Figure S2C). As was seen for cultures treated with TSA alone, hepatocyte marker genes were not induced (Figure 2(d) and Figure S2D), but the mesodermal markers,* MYOD1* and* FLK1*, were induced (Figure 2(e) and Figure S2E). Other mesodermal (Figure 2(e) and Figure S2E) and pluripotency marker genes (Figure 2(f) and Figure S2F) were also not induced. Again, when the epigenetic modifiers were removed, expression of the pancreatic endodermal genes was not maintained. Finally, we also demonstrated that, aside from TSA, CRM was essential for induction of endodermal and pancreatic endodermal transcripts and that induction of the pancreatic endoderm genes was more efficient when early passage cells were used. We next assessed if combining 5AZA with TSA-CRM increases the expression level of definitive endoderm and early and late pancreatic endocrine progenitor transcripts. In both cell lines, induction of most, albeit not all, endodermal and early and late pancreatic marker genes was not improved by pretreatment of the cells with 5AZA before addition of TSA-CRM (Figures S3-4A–C). 5AZA pretreatment enhanced expression of the skeletal muscle marker* MYOD1* (Figure S3E) but not hepatocyte and pluripotency marker genes (Figures S3-4D and F). Despite the morphological changes observed following treatment with 5AZA-CRM for 2 days, no induction of pancreatic or other lineage specific markers was observed (Figures S5-6).

## 4. Discussion

We demonstrated that treatment of two different sources of human fibroblasts with TSA in CRM induced expression of endoderm lineage markers and pancreatic endoderm markers known to be important for *β*-cell differentiation, albeit at modest levels. By contrast, 5AZA did not induce expression of endoderm and pancreatic endoderm markers though morphological changes occurred. However, the induction resulting from treatment with TSA, or a combination of 5AZA followed by TSA, was reversible and disappeared upon removal of the epigenetic modifiers and the chromatin remodeling medium. Interestingly, already in 1977, Taylor and Johns demonstrated that 5AZA can support transdifferentiation of mouse fibroblasts into skeletal myoblasts by the master regulator* MyoD* [29, 30] but did not recognize the involvement of epigenetics.

In line with our studies, Pennarossa et al. [19] described that culture of human fibroblasts with 5AZA resulted in minimal induction of endodermal and pancreatic marker genes. However, they demonstrated that such pretreatment allows cells subsequently to differentiate into *β*-cells when cultured in the presence of cocktails of growth factors to induce pancreatic endoderm and subsequently mature endocrine pancreatic cells. In a second study by the same group [20], similar results were reported for pig fibroblasts, where again they described that treatment with 5AZA alone does not cause robust changes in endoderm and pancreatic gene expression patterns but may change the epigenetic state of fibroblasts such that they subsequently become susceptible to growth factors that support pancreatic endocrine and *β*-cell differentiation.

Another approach, described by Katz et al., combined the use of TF overexpression followed by treatment of the transduced cells on day 3 with a HDACi (Romidepsin) and 5AZA to transdifferentiate human dermal fibroblasts into *β*-cells [21]. They demonstrated that Romidepsin could activate some endocrine pancreas genes, whereas 5AZA alone did not. This corroborates our observations that the HDAC inhibitor TSA can induce endoderm and pancreatic marker genes. They further found that* PDX1* was only minimally induced despite the fact that the combination of Romidepsin and 5AZA could induce a number of markers of pancreatic endoderm and more mature pancreatic cells. Only when overexpression of* PDX1* was combined with the epigenetic modifiers, human fibroblasts could be reprogrammed to *β*-cells.

We speculate that TSA combined with CRM may induce a “versatile” epigenome surrounding endodermal and pancreatic genes but cannot induce full reprogramming. Full reprogramming may require that we combine this approach with transduction of a limited number of key pancreatic TFs (like* PDX1*) or culture in growth factor cocktails known to allow differentiation of PSC to *β*-cells. Aside from the HDACi, TSA, CRM was also needed to induce the endodermal and pancreatic genes. Which factor from CRM has this added epigenetic effect is currently still unclear, but it might be L-Ascorbic Acid, as this compound has been shown to enhance reprogramming via the induction of ten-eleven translocation (TET) hydroxylase dependent DNA demethylation [31–33].

## 5. Conclusion

In summary, we described an optimized schedule for treatment of human fibroblasts with epigenetic modifiers to activate endocrine pancreatic marker genes. We demonstrated that culture of adult human fibroblasts with TSA combined with CR medium induces expression of endoderm and pancreatic endoderm genes but that this is a reversible phenomenon. Treatment with TSA/CRM also induced some mesoderm lineage markers* MYOD1* and* FLK1* in both fibroblast cell lines. When treatment with 5AZA-CRM was added before TSA-CRM treatment, an increase in mesoderm lineage markers* MYOD1* was seen beyond levels induced by TSA-CRM, but 5AZA/CRM itself did not induce any lineage marker expression. Hence, further maintenance in *β*-cell-specifying conditions with or without forced expression of exogenous TFs will be needed to eventually convert fibroblasts to *β*-cells.

## Additional Points

1. Trichostatin A (TSA) treated fibroblasts with chromatin remodeling medium (CRM) are induced towards definitive and pancreatic endoderm cell fate.
2. TSA-CRM did not affect expression of hepatocyte and pluripotency markers but induced mesoderm lineage markers, especially* MYOD1* and* FLK1*.
3. Pretreatment of fibroblasts with 5AZA-CRM prior to TSA-CRM did not induce further increase in gene expression over levels induced with TSA-CRM alone for all markers tested.

## Supplementary Material

These supplementary figures associated with 3.Results section, 3.1 and 3.2 of the article as following order:
Figure S1: Trichostatin-A (TSA) treatment of BJ foreskin fibroblast induces transient definitive endoderm and pancreatic endoderm markers.
Figure S2: 5-azacytidine (5AZA) and Trichostatin-A (TSA) treatment of BJ foreskin fibroblast induces transient definitive endoderm and pancreatic endoderm markers.
Figure S3: Comparison of gene expression changes in primary adult fibroblasts treated with TSA-CRM vs. 5AZA-TSA-CRM.
Figure S4: Comparison of gene expression changes in BJ1 foreskin fibroblasts treated with TSA-CRM vs. 5AZA-TSA-CRM.
Figure S5: 5-azacytidine (5AZA) treatment of primary adult fibroblasts does not induce marker expression.
Figure S6: 5-azacytidine (5AZA) treatment of BJ foreskin adult fibroblast does not induces marker expression.

## Figures and Tables

**Table 1:** Primers sets used for gene expression analysis by qRT-PCR.

Gene	Forward	Reverse
*GAPDH*	TCAAGAAGGTGGTGAAGCAGG	ACCAGGAAATGAGCTTGACAAA
*GATA4*	TCCAAACCAGAAAACGGAAG	CTGTGCCCGTAGTGAGATGA
*SOX7*	GCCTGTGCAACAAGAGTGAA	GTACCCTGGGTCTTTGGTCA
*MIXL1*	GGATCCAGGTATGGTTCCAG	CATGAGTCCAGCTTTGAACC
*GSC*	TCTCAACCAGCTGCACTGTC	CCAGACCTCCACTTTCTCCTC
*EOMES*	AACAACACCCAGATGATAGTC	TCATAGTTGTCTCTGAAGCCT
*E-CADHERIN*	CGAACTATATTCTTCTGTGAGAGG	GATAGATTCTTGGGTTGGGTC
*SOX17*	CGCTTTCATGGTGTGGGCTAAGGACG	TAGTTGGGGTGGTCCTGCATGTGCTG
*FOXA2*	AGGAGGAAAACGGGAAAGAA	GGTGCTTGAAGAAGCAGGAG
*CXCR4*	CACCGCATCTGGAGAACCA	GCCCATTTCCTCGGTGTAGTT
*HNF1B*	TCACAGATACCAGCAGCATCAGT	GGGCATCCCAGGCTTGTA
*SOX9*	ACGCCATCTTCAAGGCGCTG	CCGGCTGCACGTCGGTTTT
*PTF1A*	ACGACTTCTTCACCGACCAG	TGGTGGCTAAGGAACTCCAC
*PDX1*	TCCACCTTGGGACCTGTTTA	GTGTGTTAGGGAGCCTTCCA
*HLXB9*	ATGATCCTGCCTAAGATGCC	AAATCTTCACCTGGGTCTCG
*PAX6*	CCCAAGAGCAAATTGAGGCCC	CTCTTCTCCATTTGGCCCTTCGA
*NKX6.1*	CTTCCCGTCTTTGTCCAACAA	CCATCTTCTGGCCCGGAGTGA
*NEUROD1*	TAAGACGCAGAAGCTGTCCA	CTGCTCAGGCAGAAAAGTCC
*ISL1*	GTACAACCACCATTTCACTG	CCCGTACAACCTGATATAATCTC
*ARX*	ACAGCGTGTGCCTCTCTGC	TCGGGCCTCGGTCAAGTCC
*PAX4*	CAACCGAGTCCTGCGGGCAT	GCCAGCTTTCCACGGGCCAC
*NGN3*	TCTCTATTCTTTTGCGCCGG	CTTGGACAGTGGGCGCAC
*MAFB*	GCCTGCGCTAATTGTAGGAG	CAAAAGCAGGGAAAGAAACG
*MAFA*	TCATCCGGCTCAAGCAGAAG	TCTCGCTCTCCAGAATGTGC
*INS*	ATCAAGCACATCACTGTCCT	TGTAGAAGAAGCCTCGTTCC
*SST*	GAGGCTTGAGCTGCAGAGAT	TCGCTGAAGACTTGGAGGAT
*GCG*	GTTCCCTTCAAGACACAGAG	GGCAATGTTATTCCTGTTCC
*MYOD1*	CGACGGCATGATGGACTACA	TAGTAGGCGCCTTCGTAGCA
*TIE2*	TGCCCAGATATTGGTGTCCT	CTCATAAAGCGTGGTATTCACGTA
*FLK1*	ACAACCAGACGGACAGTGGT	AGCCTTCAGATGCCACAGAC
*VE-CADHERIN*	GTTCACGCATCGGTTGTTC	TCTGCATCCACTGCTGTCA
*ALB*	ATGCTGAGGCAAAGGATGTC	AGCAGCAGCACGACAGAGTA
*AFP*	TGAGCACTGTTGCAGAGGAG	GTGGTCAGTTTGCAGCATTC
*HNF4A*	ACTACGGTGCCTCGAGCTGT	GGCACTGGTTCCTCTTGTCT
*OCT4*	GATGGCGTACTGTGGGCCC	TGGGACTCCTCCGGGTTTTG
*SOX2*	TGGCGAACCATCTCTGTGGT	CCAACGGTGTCAACCTGCAT
*NANOG*	CCTGTGATTTGTGGGCCTG	GACAGTCTCCGTGTGAGGCAT
