# Supplementary material for: Epigenetic Induction of Definitive and Pancreatic Endoderm Cell Fate in Human Fibroblasts
Source: Stem Cells Int. 2016 Jun 15;2016:7654321. doi: 10.1155/2016/7654321 (PMC4925994; doi:10.1155/2016/7654321)
Supplement: Supplementary file 1 — These supplementary figures associated with 3.Results section, 3.1 and 3.2 of the article as following order: Figure S1: Trichostatin-A (TSA) treatment of BJ foreskin fibroblast induces transient definitive endoderm and pancreatic endoderm markers. Figure S2: 5-azacytidine (5AZA) and Trichostatin-A (TSA) treatment of BJ foreskin fibroblast induces transient definitive endoderm and pancreatic endoderm markers. Figure S3: Comparison of gene expression changes in primary adult fibroblasts treated with TSA-CRM vs. 5AZA-TSA-CRM. Figure S4: Comparison of gene expression changes in BJ1 foreskin fibroblasts treated with TSA-CRM vs. 5AZA-TSA-CRM. Figure S5: 5-azacytidine (5AZA) treatment of primary adult fibroblasts does not induce marker expression. Figure S6: 5-azacytidine (5AZA) treatment of BJ foreskin adult fibroblast does not induces marker expression. [file 7654321.f1.pdf]

Supplementary Figures and Tables

Epigenetic induction of definitive and pancreatic endoderm cell fate in human fibroblasts

Table of contents

Supplementary figure 1. Trichostatin-A (TSA) treatment of BJ foreskin fibroblast induces transient definitive endoderm and pancreatic endoderm markers……………………..**2**

Supplementary figure 2. 5-azacytidine (5AZA) and Trichostatin-A (TSA) treatment of BJ foreskin fibroblast induces transient definitive endoderm and pancreatic endoderm markers…………………………………………………………………………...**3**

Supplementary figure 3. Comparison of gene expression changes in primary adult fibroblasts treated with TSA-CRM vs. 5AZA-TSA-CRM……………………………**4**

Supplementary figure 4. Comparison of gene expression changes in BJ1 foreskin fibroblasts treated with TSA-CRM vs. 5AZA-TSA-CRM……………………………**5**

Supplementary figure 5. 5-azacytidine (5AZA) treatment of primary adult fibroblasts does not induce marker expression……………………………………………………**6**

Supplementary figure 6. 5-azacytidine (5AZA) treatment of BJ foreskin adult fibroblast does not induces marker expression……………………………………………………**7**

Supplementary table-1

Primers sets used for gene expression analysis by qRT-PCR…………………………**8**

Supplementary table-2

Primers sets used for gene expression analysis by qRT-PCR…………………………..**9**

Supplementary figure-1

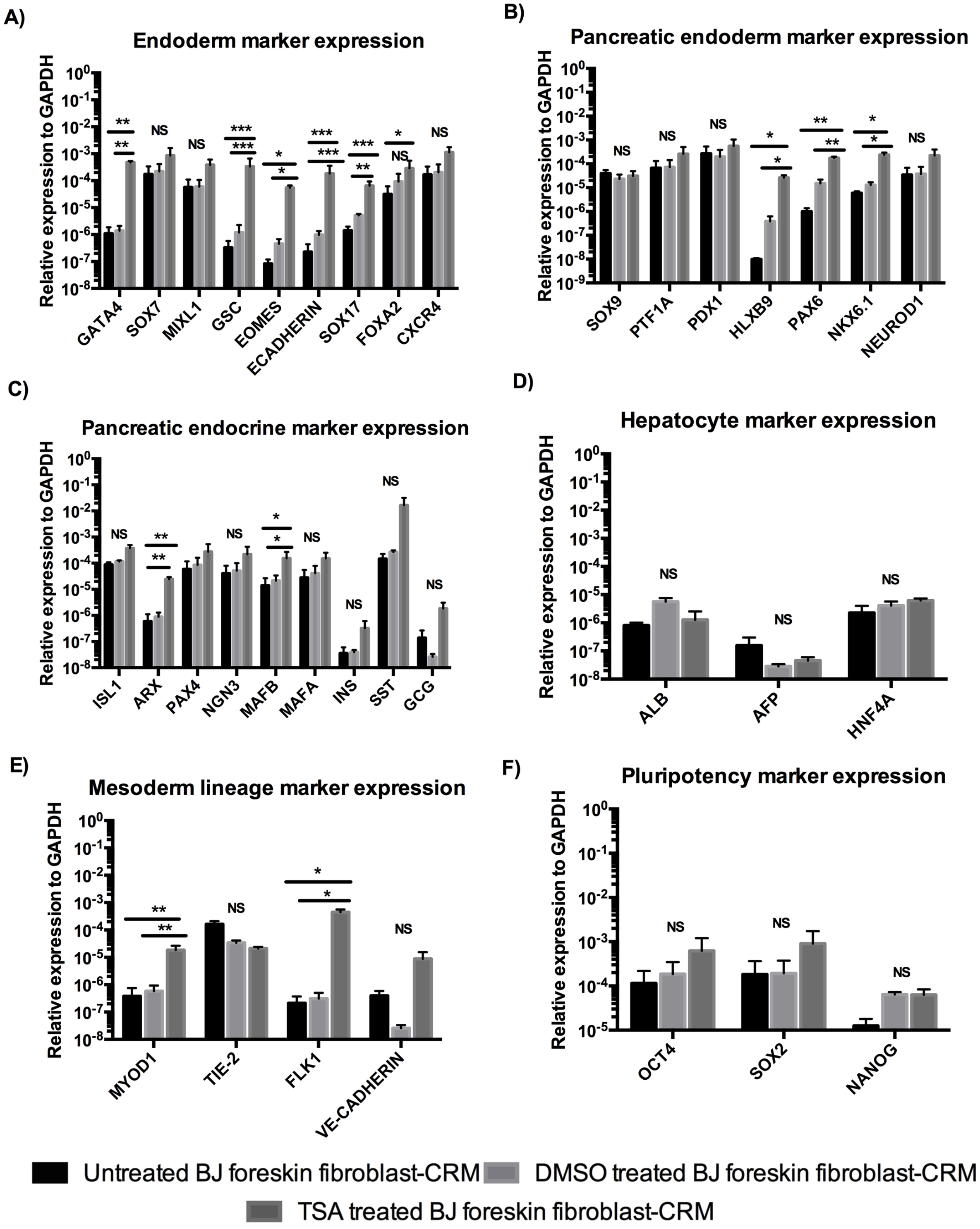

Supplementary figure-2

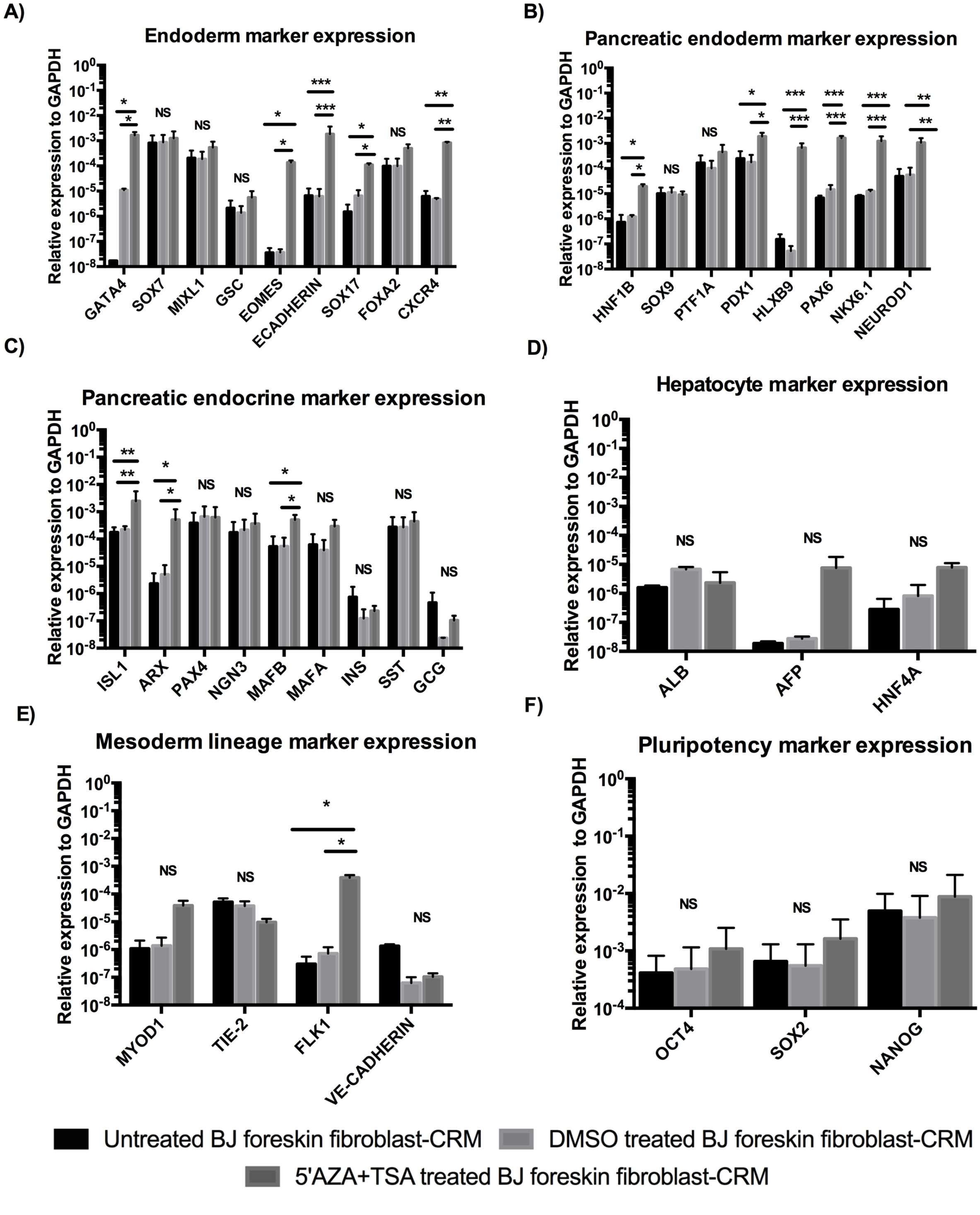

Supplementary figure-3

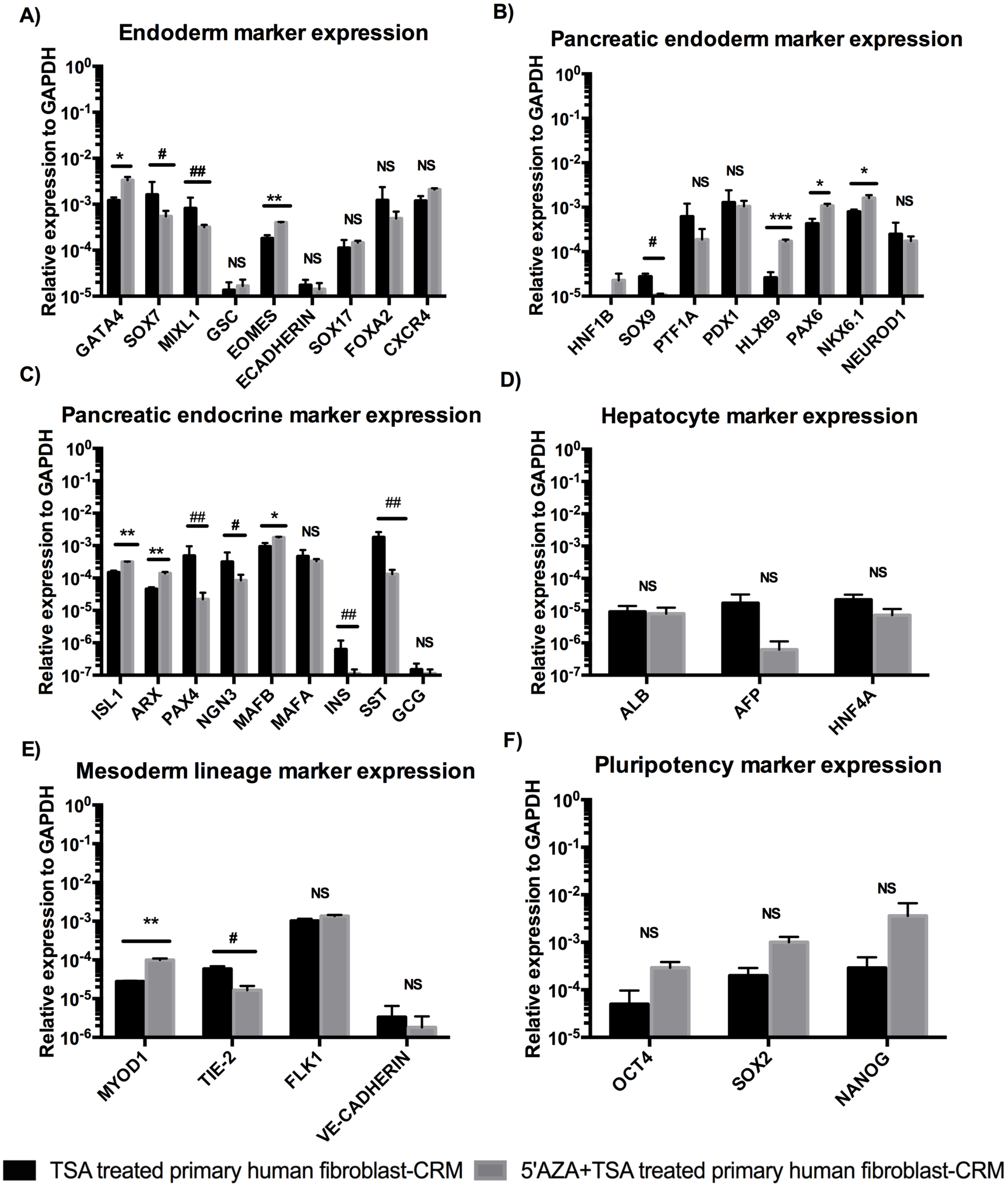

Supplementary figure-4

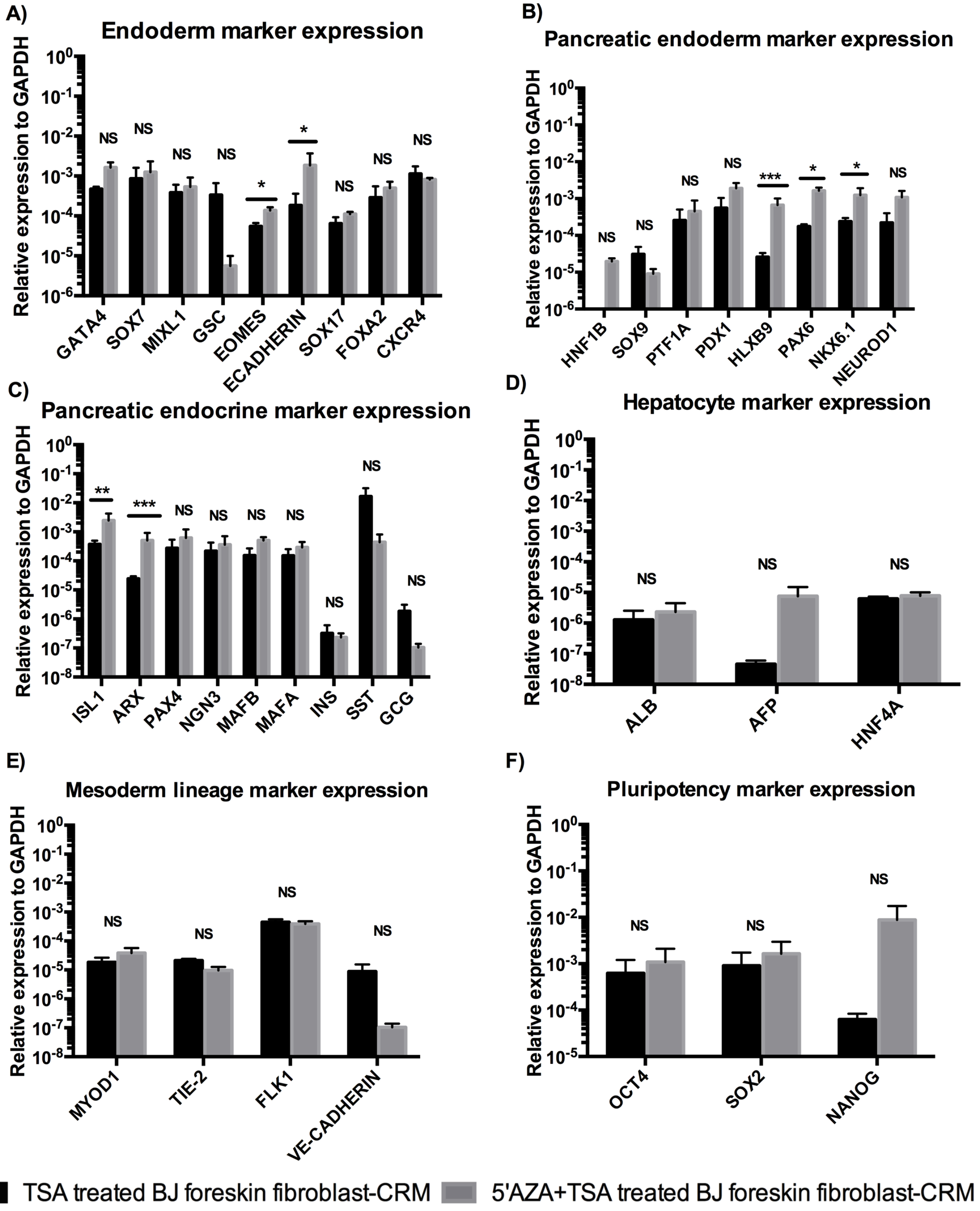

Supplementary figure-5

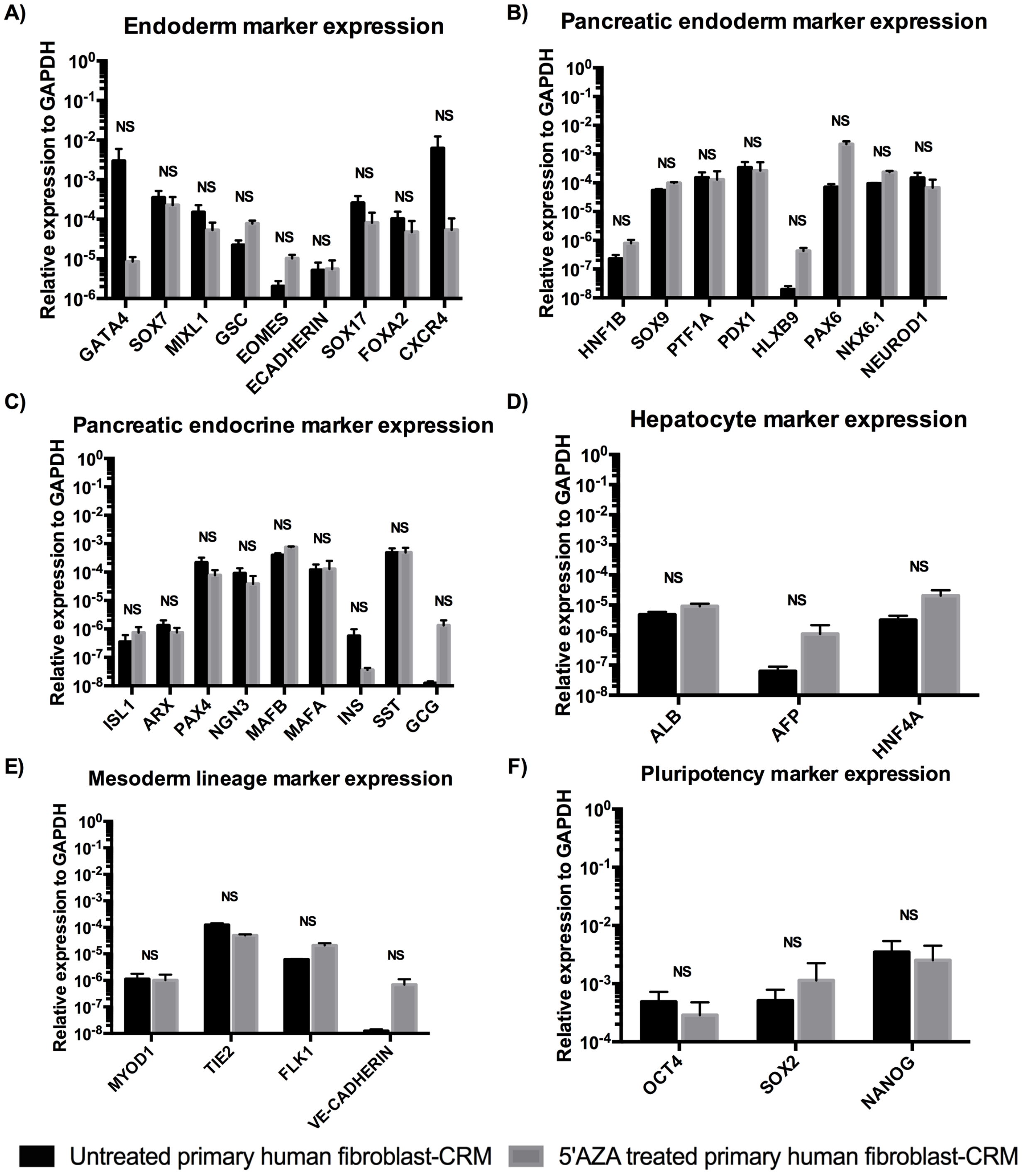

Supplementary figure-6

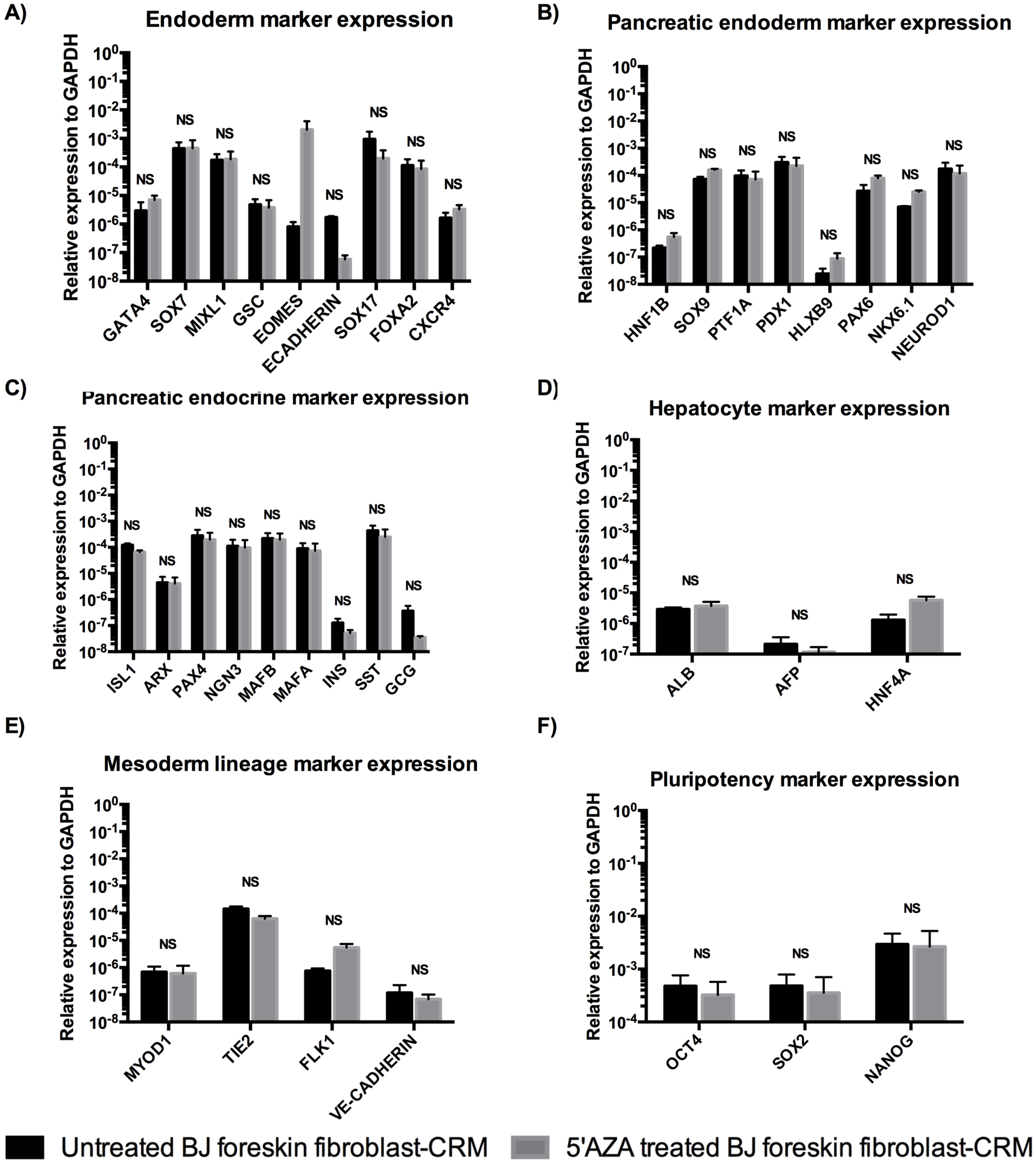

Supplementary table-1

Primers sets used for gene expression analysis by qRT-PCR

Gene	Forward	Reverse
GAPDH	TCAAGAAGGTGGTGAAGCAGG	ACCAGGAAATGAGCTTGACAAA
GATA4	TCCAAACCAGAAAACGGAAG	CTGTGCCCGTAGTGAGATGA
SOX7	GCCTGTGCAACAAGAGTGAA	GTACCCTGGGTCTTTGGTCA
MIXL1	GGATCCAGGTATGGTTCCAG	CATGAGTCCAGCTTTGAACC
GSC	TCTCAACCAGCTGCACTGTC	CCAGACCTCCACTTTCTCCTC
EOMES	AACAACACCCAGATGATAGTC	TCATAGTTGTCTCTGAAGCCT
ECADHERIN	CGAACTATATTCTTCTGTGAGAGG	GATAGATTCTTGGGTTGGGTC
SOX17	CGCTTTCATGGTGTGGGCTAAGGACG	TAGTTGGGGTGGTCCTGCATGTGCTG
FOXA2	AGGAGGAAAACGGGAAAGAA	GGTGCTTGAAGAAGCAGGAG
CXCR4	CACCGCATCTGGAGAACCA	GCCCATTTCCTCGGTGTAGTT
HNF1B	TCACAGATACCAGCAGCATCAGT	GGGCATCCCAGGCTTGTA
SOX9	ACGCCATCTTCAAGGCGCTG	CCGGCTGCACGTCGGTTTT
PTF1A	ACGACTTCTTCACCGACCAG	TGGTGGCTAAGGAACTCCAC
PDX1	TCCACCTTGGGACCTGTTTA	GTGTGTTAGGGAGCCTTCCA
HLXB9	ATGATCCTGCCTAAGATGCC	AAATCTTCACCTGGGTCTCG
PAX6	CCCAAGAGCAAATTGAGGCCC	CTCTTCTCCATTTGGCCCTTCGA
NKX6.1	CTTCCCGTCTTTGTCCAACAA	CCATCTTCTGGCCCGGAGTGA
NEUROD1	TAAGACGCAGAAGCTGTCCA	CTGCTCAGGCAGAAAAGTCC
ISL1	GTACAACCACCATTTCACTG	CCCGTACAACCTGATATAATCTC
ARX	ACAGCGTGTGCCTCTCTGC	TCGGGCCTCGGTCAAGTCC
PAX4	CAACCGAGTCCTGCGGGCAT	GCCAGCTTTCCACGGGCCAC

Supplementary table-2

Primers sets used for gene expression analysis by qRT-PCR

**Gene**	**Forward primer (5'-3')**	**Reverse primer (3'-5')**
NGN3	TCTCTATTCTTTTGCGCCGG	CTTGGACAGTGGGCGCAC
MAFB	GCCTGCGCTAATTGTAGGAG	CAAAAGCAGGGAAAGAAACG
MAFA	TCATCCGGCTCAAGCAGAAG	TCTCGCTCTCCAGAATGTGC
INS	ATCAAGCACATCACTGTCCT	TGTAGAAGAAGCCTCGTTCC
SST	GAGGCTTGAGCTGCAGAGAT	TCGCTGAAGACTTGGAGGAT
GCG	GTTCCCTTCAAGACACAGAG	GGCAATGTTATTCCTGTTCC
MYOD1	CGACGGCATGATGGACTACA	TAGTAGGCGCCTTCGTAGCA
TIE2	TGCCCAGATATTGGTGTCCT	CTCATAAAGCGTGGTATTCACGTA
FLK1	ACAACCAGACGGACAGTGGT	AGCCTTCAGATGCCACAGAC
VE-CADHERIN	GTTCACGCATCGGTTGTTC	TCTGCATCCACTGCTGTCA
ALB	ATGCTGAGGCAAAGGATGTC	AGCAGCAGCACGACAGAGTA
AFP	TGAGCACTGTTGCAGAGGAG	GTGGTCAGTTTGCAGCATTC
HNF4A	ACTACGGTGCCTCGAGCTGT	GGCACTGGTTCCTCTTGTCT
OCT4	GATGGCGTACTGTGGGCCC	TGGGACTCCTCCGGGTTTTG
SOX2	TGGCGAACCATCTCTGTGGT	CCAACGGTGTCAACCTGCAT
NANOG	CCTGTGATTTGTGGGCCTG	GACAGTCTCCGTGTGAGGCAT
